# Confirmation of Metformin-Induced Acute Generalized Exanthematous Pustulosis Through a Positive Dechallenge-Rechallenge Test: A Case Report and Review of Cutaneous Manifestations Associated With Metformin

**DOI:** 10.7759/cureus.77441

**Published:** 2025-01-14

**Authors:** Haifa A Alfalah, Khalid N Nagshabandi

**Affiliations:** 1 Department of Internal Medicine and Critical Care, King Abdullah Bin Abdulaziz University Hospital, Riyadh, SAU; 2 Department of Dermatology, College of Medicine, King Saud University Medical City, Riyadh, SAU

**Keywords:** acute generalized exanthematous pustulosis, antidiabetic, cutaneous adverse drug reaction, dechallenge-rechallenge test, exanthematous drug eruption, metformin

## Abstract

Metformin, a widely used first-line treatment for type 2 diabetes mellitus (T2DM), is known for its efficacy and safety profile. While gastrointestinal disturbances are the most commonly reported with metformin use, dermatologic reactions, particularly severe ones, are rare and underrecognized. We present a case of a 45-year-old male with a history of atrial fibrillation who developed acute generalized exanthematous pustulosis (AGEP) after initiating metformin for prediabetes.

## Introduction

Metformin, a cornerstone of diabetes management, belongs to the biguanide class of oral hypoglycemic agents, a class known for its unique mechanism of action in lowering blood glucose levels. Biguanides primarily work by reducing hepatic glucose production through the inhibition of gluconeogenesis, thereby addressing a key pathological feature of type 2 diabetes mellitus (T2DM). This mechanism, coupled with metformin’s ability to improve insulin sensitivity in peripheral tissues such as skeletal muscle, sets it apart from other antidiabetic medications that act by stimulating insulin secretion. Since its introduction in the 1950s, metformin has become one of the most widely prescribed antidiabetic medications globally due to its efficacy, affordability, and favorable safety profile [1]. Unlike other antidiabetic agents, metformin is associated with a minimal risk of hypoglycemia, which has contributed to its widespread acceptance [1]. Despite its well-established benefits, metformin is not without adverse effects. Gastrointestinal disturbances are the most commonly reported side effects [2]. However, cutaneous adverse drug reactions (CADRs) are rare and underrecognized, particularly severe manifestations such as acute generalized exanthematous pustulosis (AGEP).

AGEP is a rare and severe skin condition characterized by the abrupt onset of numerous sterile pustules on a background of erythema. This condition most commonly arises as an adverse reaction to certain medications, particularly antibiotics such as beta-lactams and macrolides. Although less frequent, AGEP can also be triggered by viral infections or exposure to heavy metals. Clinically, AGEP manifests with a sudden, widespread eruption of pustules, typically accompanied by fever and an increase in white blood cell count, often occurring within hours to a few days after exposure to the triggering agent [3]. Drug eruptions induced by metformin are a subset of CADRs, which encompass a wide range of skin manifestations, from mild exanthematous to severe and potentially life-threatening rashes [4]. Dermatological reactions from using metformin, although rare and uncommon, are of significant clinical interest and concern. Given the increasing prevalence of diabetes and the widespread use of metformin, awareness of its potential to cause drug eruptions is crucial. Early identification and appropriate management of these adverse reactions can prevent further serious complications. Herein, we report a novel instance of a 45-year-old prediabetic male patient who developed AGEP after commencing metformin therapy, biopsy-proven and further confirmed with a positive dechallenge-rechallenge test.

## Case presentation

**Figure 1:**
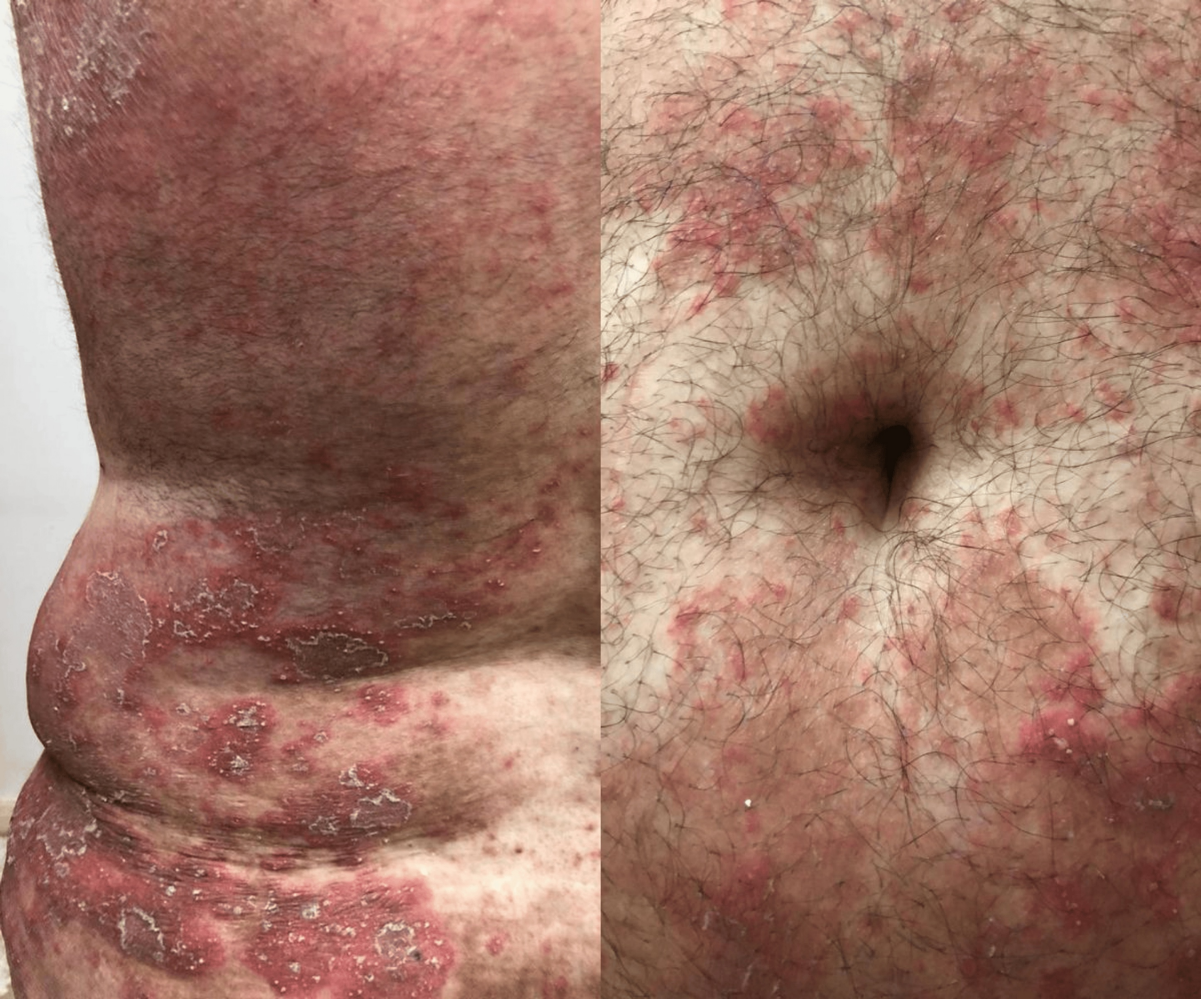
Multiple scaly erythematous plaques and patches exhibiting numerous superficial non-follicular pustules on the back and periumbilical abdominal area. The lesions are well-demarcated, with a clear distinction between affected and unaffected skin.

**Figure 2:**
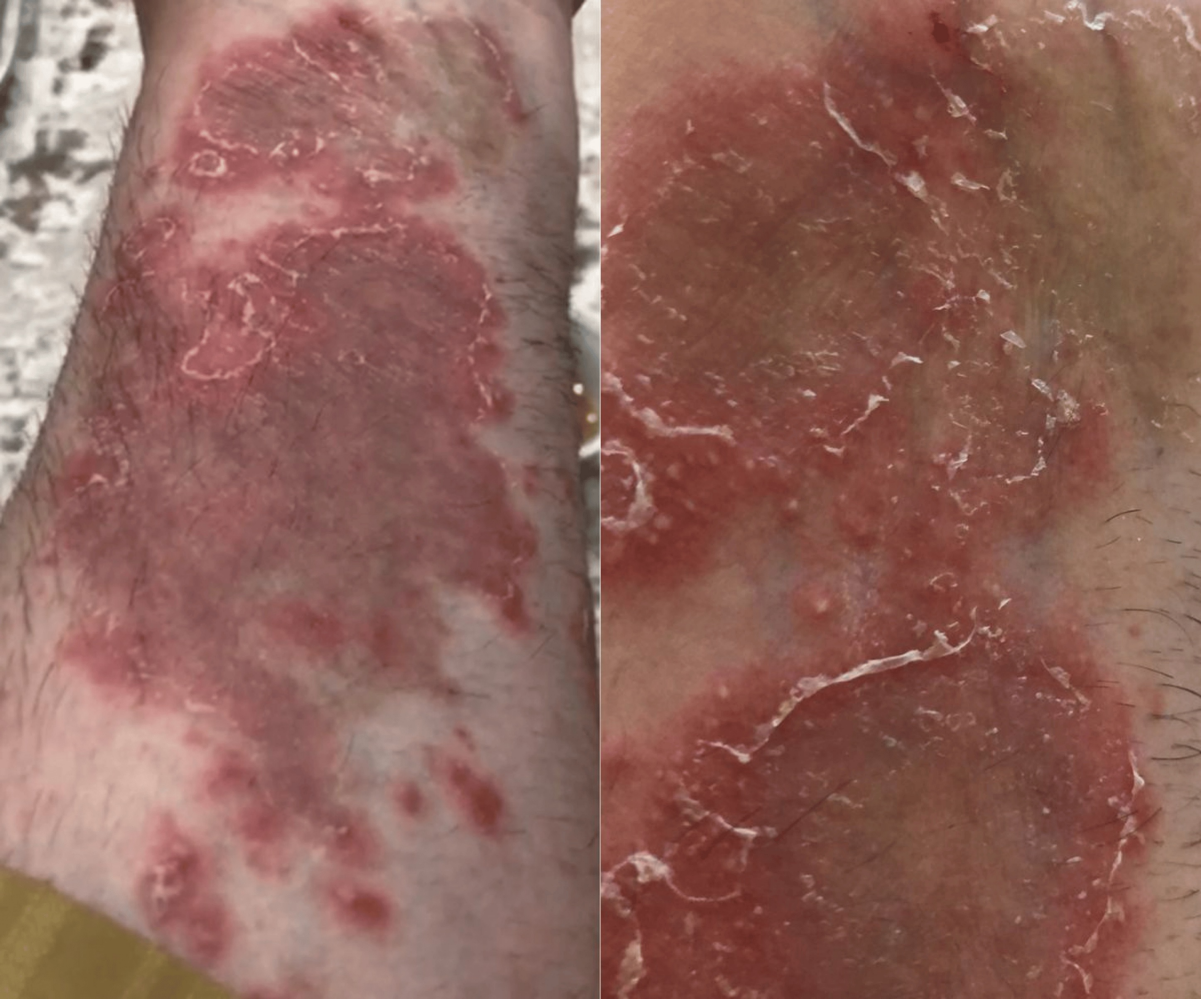
Irregularly shaped erythematous plaques with a scaly pustular eruption on the dorsal aspect of the bilateral forearms and wrists.

**Figure 3:**
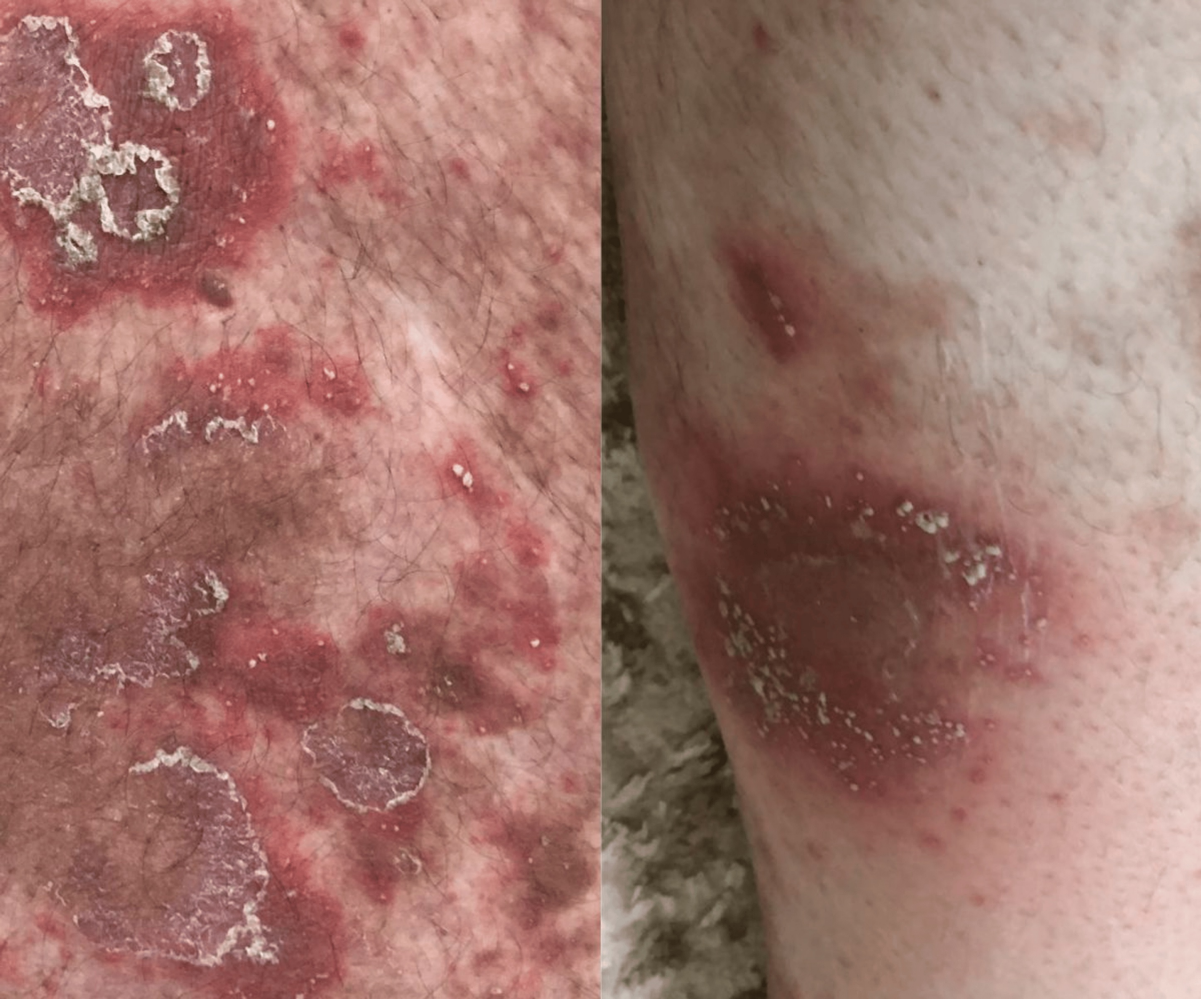
Erythematous scaly plaques, with overlying non-follicular pustules scattered across the thighs and legs, with some areas of confluent erythema.

**Figure 4:**
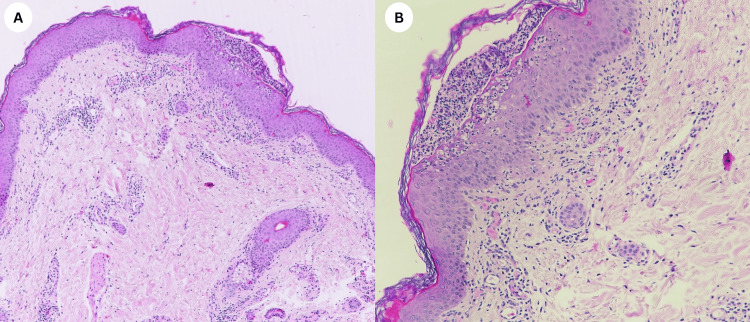
(A, B) Histopathological examination revealed subcorneal pustules with epidermal spongiosis and neutrophil infiltrate within the upper epidermis. Along with mild perivascular inflammatory infiltrate in the superficial dermis, predominantly composed of neutrophils and scattered eosinophils. The overlying stratum corneum appears disrupted in areas, consistent with pustular exanthema. (A) (H/E stain, original magnification x10), (B) (H/E stain, original magnification x20).

A 45-year-old obese male with a medical history significant for atrial fibrillation managed with apixaban for 10 years, was recently diagnosed with prediabetes by his family physician. His HbA1C level (6.3%) was found to be elevated, prompting the initiation of metformin therapy at a dose of 500 mg once daily. Three days after starting metformin, the patient presented to the dermatology outpatient clinic with a diffuse, erythematous skin rash covering his entire body. Physical examination revealed numerous small, non-follicular pustules within a large background of scaly erythematous and edematous patches and plaques involving the back, abdomen, chest (Figure 1), and upper and lower extremities (Figures 2-3). The lesions were not pruritic or painful. Notably, there was no involvement of the mucous membranes, palms, or soles, and there were no blisters, skin sloughing, eye changes, or target lesions, and the Nikolsky sign was negative. Given the clinical presentation and suspicion of AGEP, the patient was advised to discontinue metformin immediately. He was prescribed topical Clobetasol propionate 0.05% to be applied over the affected areas twice daily for two weeks. For the face, inframammary, and inguinal folds, a combination of topical hydrocortisone 1% and miconazole nitrate 2% was recommended twice daily for two weeks. Frequent skin moisturization and oral antihistamines were also prescribed. To further investigate the diagnosis, a 5-mm punch skin biopsy was performed. Histopathological examination of the biopsy specimen revealed subcorneal pustules with surrounding spongiosis and superficial perivascular inflammatory infiltrates predominantly consisting of neutrophils and eosinophils, consistent with AGEP (Figure 4). PAS special stain was negative for fungal organisms. Based on clinicopathological correlation and exclusion of any other potential causes, the diagnosis of AGEP was made. The patient was instructed to follow up with his family physician for an alternative medication to metformin. Remarkably, the patient noted significant improvement within four days of discontinuing metformin and starting the prescribed topical therapy.

**Figure 5:**
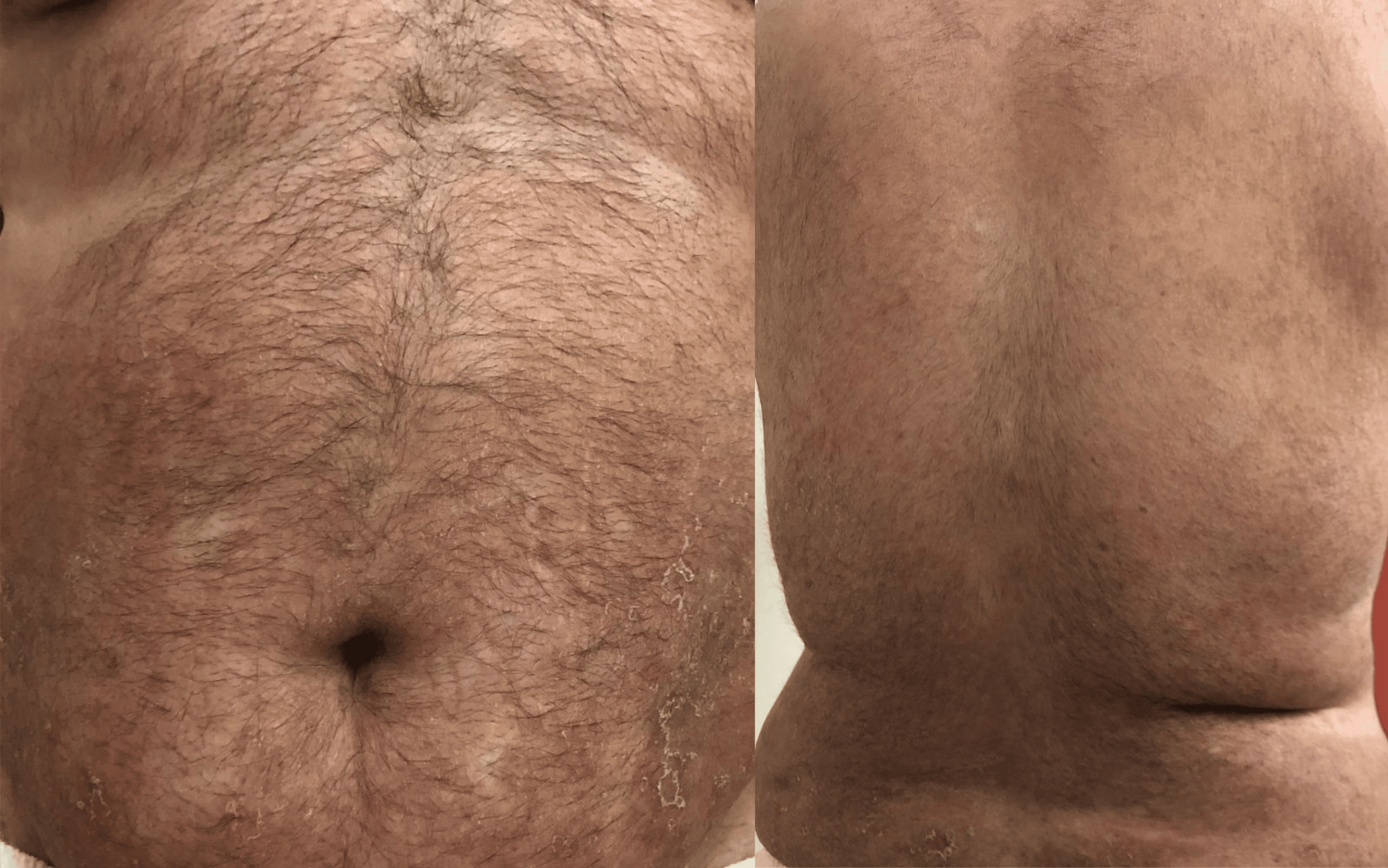
Generalized exfoliation and marked improvement after drug discontinuation with a significant reduction in erythema and scaling. The pustules resolved completely, leaving areas of post-inflammatory hyperpigmentation and mild residual scaling on previously affected skin.

**Figure 6:**
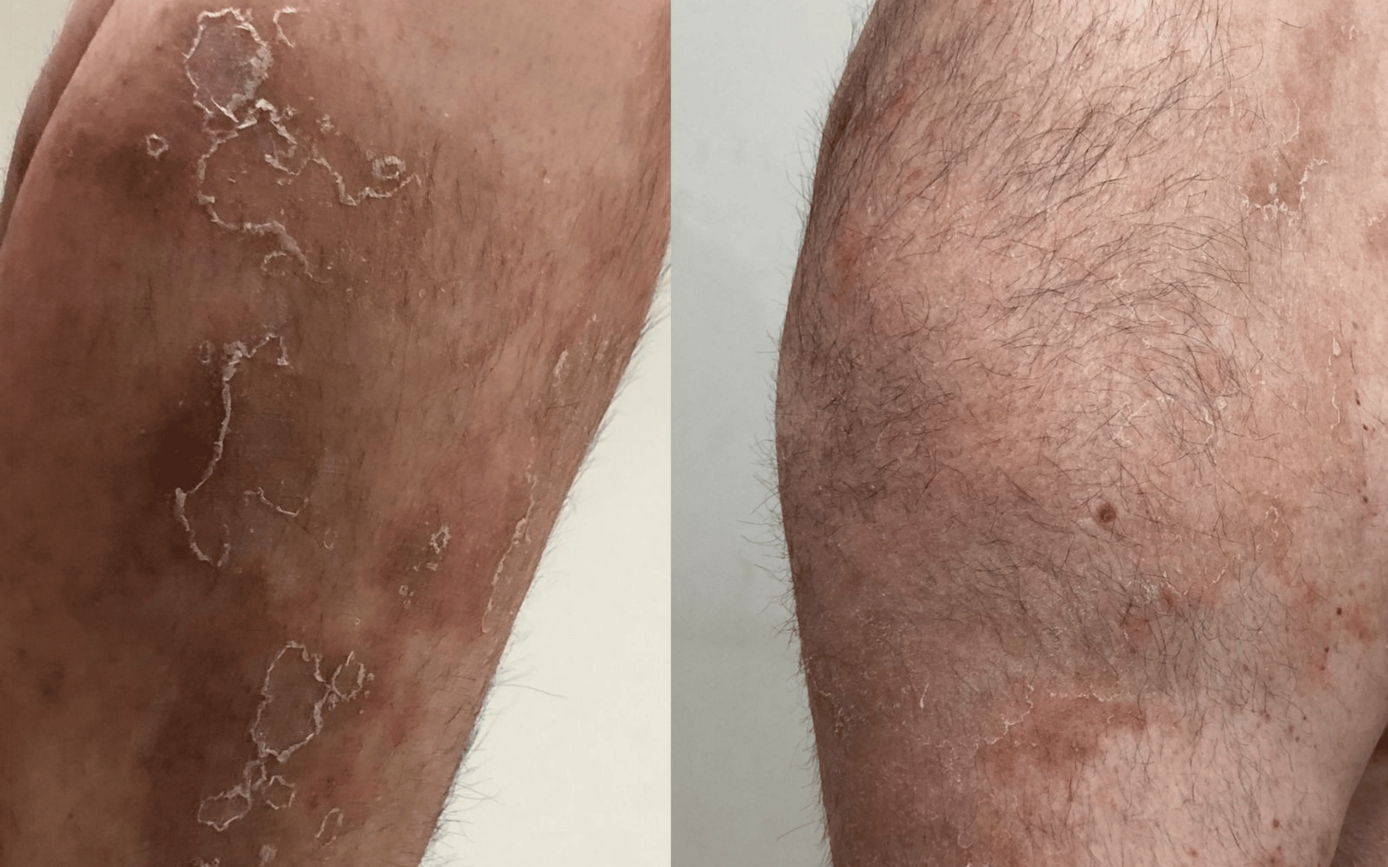
Generalized exfoliation and marked improvement after drug discontinuation with a significant reduction in erythema and scaling. The pustules resolved completely, leaving areas of post-inflammatory hyperpigmentation and mild residual scaling on previously affected skin.

Approximately six weeks after discontinuing metformin, the patient was restarted on the medication, albeit a different brand, by his family physician. Four days after resuming metformin, the patient noticed the reappearance of the pustular rash and immediately discontinued the medication. He returned to the dermatology clinic two weeks after restarting metformin. On examination, generalized exfoliation and resolution of erythema and pustules were noted in the previously affected areas, with signs of post-inflammatory hyperpigmentation indicating recovery from the recurrent AGEP (Figures 5-6). The recurrence of the rash following re-exposure to metformin, coupled with its resolution upon cessation, confirmed the diagnosis of metformin-induced AGEP through a positive dechallenge-rechallenge test.

## Discussion

Metformin is a well-tolerated oral medication that belongs to the biguanide class of drugs, primarily used to manage T2DM. It was first introduced in the 1950s and has since become one of the most widely prescribed antidiabetic drugs globally due to its efficacy, safety profile, and cost-effectiveness [1]. Metformin primarily lowers blood glucose levels by reducing hepatic glucose production through the inhibition of gluconeogenesis. It achieves this by activating the enzyme AMP-activated protein kinase (AMPK), a critical regulator of energy balance within cells. Additionally, metformin enhances insulin sensitivity in peripheral tissues, particularly in skeletal muscles, which facilitates increased glucose uptake and utilization [1]. Unlike other antidiabetic medications, metformin does not stimulate insulin secretion, which significantly reduces the risk of hypoglycemia, making it safer for long-term use [1,5].

Over time, research has unveiled additional benefits of metformin for various diseases, including cancers, cardiovascular diseases, liver diseases, and even anti-aging neuroprotective effects [1]. Metformin’s ability to inhibit platelet aggregation and act as an antioxidant has made it an effective therapeutic solution in non-diabetic settings, including dermatologic conditions such as hirsutism, acne, hidradenitis suppurativa, acanthosis nigricans, psoriasis, and skin cancer like melanoma [1,6]. Its use has shown promising outcomes in influencing melanoma growth and metastasis through several mechanisms, such as induction of cell cycle arrest, inhibition of TRB3 expression, activation of AMPK, and potential synergy with other therapies like combining metformin alongside other immunotherapies, anti-cytotoxic T-lymphocyte antigen 4 (CTLA-4) (ipilimumab) and anti-PD-1 (nivolumab), which are already approved for melanoma treatment [1].

In addition to metformin’s benefit in various cutaneous conditions, it can also cause multiple reported dermatologic reactions and manifestations, including pruritus, hyperhidrosis, non-specific rash, photosensitivity, leukocytoclastic vasculitis, bullous pemphigoid, lichen planus, urticaria, acute alopecia, drug reaction with eosinophilia and systemic symptoms (DRESS) syndrome, as well as fixed drug eruptions and exanthematous immunologic CADRs such as blistering, psoriasiform, generalized macular, generalized bullous, rosacea-like facial rash, and lichenoid eruptions [4,6-20]. Our case highlights a novel clinical instance of AGEP induced by the use of metformin and confirmed with a dechallenge-rechallenge test. Other antidiabetic drugs such as sulfonylureas, meglitinides, thiazolidinediones, and insulin can induce a multitude of cutaneous manifestations and adverse effects that can range from benign non-specific rash to severe life-threatening reactions such as Stevens-Johnson syndrome (SJS) and toxic epidermal necrolysis (TEN) [4].

AGEP is marked by the sudden appearance of numerous small, sterile, non-follicular pustules on inflamed erythematous skin, typically within 24-48 hours after exposure to a triggering drug. The condition is more frequently observed in women and has an incidence of one to five cases per million annually. AGEP is most commonly triggered by certain medications. The drugs frequently associated with AGEP include antibiotics (beta-lactam and macrolides), antimalarials, antifungals, and calcium channel blockers [3]. Our case reports the first instance of metformin-induced AGEP. Histopathological characteristics of AGEP lesions include subcorneal or intraepidermal pustules with surrounding spongiosis and superficial perivascular inflammatory cells infiltrated with lymphocytes, neutrophils, and eosinophils [3]. In our case, the diagnosis of AGEP was confirmed with both a skin biopsy and a positive dechallenge-rechallenge test, supporting the strong temporal association with drug exposure. The findings, observed in (Figure 4), were consistent with AGEP. Moreover, the diagnosis was further confirmed through a dechallenge-rechallenge test. Upon discontinuation of the suspected medication, the patient's symptoms rapidly improved, demonstrating a positive dechallenge. The medication was reintroduced, which resulted in the recurrence of the characteristic pustular rash, thereby confirming the diagnosis of AGEP with a positive rechallenge response. The primary treatment for AGEP is the immediate discontinuation of the suspected drug. Topical corticosteroids are the first line of treatment, and systemic corticosteroids may be used in more severe cases [3].

## Conclusions

Dermatological reactions linked to metformin, though infrequent, warrant significant clinical attention. As diabetes prevalence rises and metformin use becomes increasingly widespread, it is crucial for clinicians to remain aware of its potential to induce drug eruptions such as AGEP. Prompt recognition and effective management of these adverse reactions are essential to avert serious complications and optimize patient outcomes.

## References

[1] Lv Z, Guo Y (2020). Metformin and its benefits for various diseases. Front Endocrinol (Lausanne).

[2] Ramu SK, Praveen Praveen, Ankith Ankith, Yadav K (2022). Study of diversity of metformin related gastrointestinal side effects. J Assoc Physicians India.

[3] Stadler PC, Oschmann A, Kerl-French K, Maul JT, Oppel EM, Meier-Schiesser B, French LE (2023). Acute generalized exanthematous pustulosis: clinical characteristics, pathogenesis, and management. Dermatology.

[4] Boccardi A, Shubrook JH (2022). Cutaneous reactions to antidiabetic agents: a narrative review. Diabetology.

[5] Nishimura R, Taniguchi M, Takeshima T, Iwasaki K (2022). Efficacy and safety of metformin versus the other oral antidiabetic drugs in Japanese Type 2 diabetes patients: a network meta-analysis. Adv Ther.

[6] Badr D, Kurban M, Abbas O (2013). Metformin in dermatology: an overview. J Eur Acad Dermatol Venereol.

[7] Voore P, Odigwe C, Mirrakhimov AE, Rifai D, Iroegbu NA (2016). DRESS syndrome following metformin administration: a case report and review of the literature. Am J Ther.

[8] Nakatani K, Kurose T, Hyo T, Watanabe K, Yabe D, Kawamoto T, Seino Y (2012). Drug-induced generalized skin eruption in a diabetes mellitus patient receiving a dipeptidyl peptidase-4 inhibitor plus metformin. Diabetes Ther.

[9] Sharma A, Baldi A, Sharma DK (2017). Drug induced generalized skin eruption in a diabetes mellitus patient receiving a metformin plus simvastatin in a tertiary care teaching hospital in Punjab. Curr Res Diabetes Obes J.

[10] Kaushal J, Rakesh A (2020). Fixed Drug Eruption: A Rare Case of Polysensitivity Between Two Unrelated Fixed Dose Combination Preparations-A Case Report.

[11] Skandalis K, Spirova M, Gaitanis G, Tsartsarakis A, Bassukas ID (2012). Drug-induced bullous pemphigoid in diabetes mellitus patients receiving dipeptidyl peptidase-IV inhibitors plus metformin. J Eur Acad Dermatol Venereol.

[12] Zaïem A, Sahnoun R, Badri T (2014). Lichen associated with metformin. Therapies.

[13] Azzam H, Bergman R, Friedman-Birnbaum R (1997). Lichen planus associated with metformin therapy. Dermatology.

[14] Sheh T, Tsai I (2016). Metformin vasculitis: a rare reaction to a common medication. Proc UCLA Healthc.

[15] Steber CJ, Perkins SL, Harris KB (2016). Metformin-induced fixed-drug eruption confirmed by multiple exposures. Am J Case Rep.

[16] Abtahi-Naeini B, Momen T, Amiri R, Rajabi P, Rastegarnasab F (2023). Metformin-induced generalized bullous fixed-drug eruption with a positive dechallenge-rechallenge test: a case report and literature review. Case Rep Dermatol Med.

[17] Ramírez-Bellver JL, Lopez J, Macias E (2017). Metformin-induced generalized fixed drug eruption with cutaneous hemophagocytosis. Am J Dermatopathol.

[18] Mumoli L, Gambardella A, Labate A, Succurro E, De Sarro G, Arturi F, Gallelli L (2014). Rosacea-like facial rash related to metformin administration in a young woman. BMC Pharmacol Toxicol.

[19] Butola LK, Meshram A, Dhok A (2020). Urticaria due to adverse drug reaction in diabetes: a case presentation. J Evolution Med Dent Sci.

[20] Koca R, Altinyazar HC, Yenidünya S, Tekin NS (2003). Psoriasiform drug eruption due to metformin hydrochloride: a case report. Dermatol Online J.

